# Evaluation of sustainable susceptibility to *Plasmodium vivax* infection among colonized *Anopheles darlingi* and *Anopheles deaneorum*

**DOI:** 10.1186/s12936-022-04204-8

**Published:** 2022-06-03

**Authors:** Najara A. C. Santos, Alice O. Andrade, Thais C. Santos, Leandro N. Martinez, Amália S. Ferreira, Alessandra S. Bastos, Mirilene M. Martins, José D. C. Pontual, Carolina B. G. Teles, Jansen F. Medeiros, Maisa S. Araújo

**Affiliations:** 1 Plataforma de Produção e Infecção de Vetores da Malária (PIVEM), Laboratório de Entomologia, Fiocruz Rondônia, Porto Velho, Rondônia Brazil; 2 Programa de Pós-Graduação em Biologia Experimental, Fundação Universidade Federal de Rondônia, FIOCRUZ Rondônia, Porto Velho, Rondônia Brazil; 3 Plataforma de Bioensaios de Malária e Leishmaniose da Fiocruz (PBML), Fiocruz Rondônia, Porto Velho, Rondônia Brazil; 4 Instituto Nacional de Epidemiologia na Amazônia Ocidental, INCT-EpiAmO, Porto Velho, Rondônia Brazil

**Keywords:** *Anopheles darlingi*, *Anopheles deaneorum*, *Plasmodium vivax*, Gametocytaemia, Colony, Susceptibility, DMFA, Survival

## Abstract

**Background:**

The colonization of mosquitoes susceptible to *Plasmodium vivax* via direct membrane feeding assay (DMFA) has the potential to significantly advance our knowledge of *P. vivax* biology, vector-parasite interaction and transmission-blocking vaccine research. *Anopheles darlingi* and *Anopheles deaneorum* are important vectors of malaria in the Western Brazilian Amazon. Since 2018, well-established colonies of these species have been maintained in order to mass produce mosquitoes destined for *P. vivax* infection. *Plasmodium* susceptibility was confirmed when the colonies were established, but susceptibility needs to be maintained for these colonies to remain good models for pathogen transmission. Thus, the susceptibility was assessed of colonized mosquitoes to *P. vivax* isolates circulating in the Western Amazon.

**Methods:**

Laboratory-reared mosquitoes from F10-F25 generations were fed on *P. vivax* blood isolates via DMFA. Susceptibility was determined by prevalence and intensity of infection as represented by oocyst load seven days after blood feeding, and sporozoite load 14 days after blood feeding. The effect of infection on mosquito survival was evaluated from initial blood feeding until sporogonic development and survival rates were compared between mosquitoes fed on infected and uninfected blood. Correlation was calculated between gametocytaemia and prevalence/intensity of infection, and between oocyst and sporozoite load.

**Results:**

Significant differences were found in prevalence and intensity of infection between species. *Anopheles darlingi* showed a higher proportion of infected mosquitoes and higher oocyst and sporozoite intensity than *An. deaneorum*. Survival analysis showed that *An. deaneorum* survival decreased drastically until 14 days post infection (dpi). *Plasmodium vivax* infection decreased survival in both species relative to uninfected mosquitoes. No correlation was observed between gametocytaemia and prevalence/intensity of infection, but oocyst and sporozoite load had a moderate to strong correlation.

**Conclusions:**

Colonized *An. darlingi* make excellent subjects for modelling pathogen transmission. On the other hand, *An. deaneorum* could serve as a model for immunity studies due the low susceptibility under current colonized conditions. In the application of DMFA, gametocyte density is not a reliable parameter for predicting mosquito infection by *P. vivax*, but oocyst intensity should be used to schedule sporozoite experiments.

**Supplementary Information:**

The online version contains supplementary material available at 10.1186/s12936-022-04204-8.

## Background

Human malaria is an infectious disease caused generally by five protozoa of the *Plasmodium* genera: *Plasmodium falciparum, Plasmodium vivax, Plasmodium ovale, Plasmodium malariae*, and *Plasmodium knowlesi*; although other *Plasmodium* species, such as *Plasmodium simium* and *Plasmodium cynomolgi*, that naturally infected non-human primates have been considered as potential threats to human health through zoonosis [1, 2]. *Plasmodium vivax* is the dominant malaria parasite in most countries outside of sub-Saharan Africa, and although it is frequently considered to be low pathogenic, it is an important cause of morbidity and mortality in endemic areas in central and South America, and in regions of Asia and Oceania. This species is responsible for the majority of malaria cases in the Brazilian Amazon [3]. The epidemiology of vivax malaria is greatly influenced by hypnozoite formation, which can cause relapses after weeks or months of treatment [4]. *Plasmodium vivax* is also more efficient at mosquito infection as it is able to maintain the transmission chain at low gametocyte densities [4–6]. This *P. vivax* profile is especially pertinent to malaria control in endemic areas [3, 6]. *Plasmodium vivax* transmission is currently controlled through vector control and access to effective treatment, but the development of new treatment and control strategies is imperative [7].

However, understanding *P. vivax* in its asexual and sexual stages has been a great challenge because *P. vivax* has been available only in non-continuous culture. The colonization of *P. vivax-*susceptible mosquitoes from endemic areas and the use of standardized direct membrane feeding assay (DMFA) have the potential to rapidly advance our knowledge of *P. vivax* biology and vector-parasite interaction, and to advance transmission-blocking vaccine (TBV) research [8].

Since 2018, two important vectors of malaria in the Western Brazilian Amazon have been maintained in laboratory colonies: *Anopheles darlingi* and *Anopheles deaneorum* [9, 10]. These well-established colonies have been maintained under standardized protocols that allow for the mass production of mosquitoes, and high levels of *P. vivax* infection. When these colonies were established, both species were tested for susceptibility to *P. vivax* via DMFA; once susceptibility was confirmed, these colonies were used to study *P. vivax*-vector interactions [9–11]. Biological, vector-parasite interaction and drug assay studies of *P. vivax* infection were conducted to answer important questions about vivax malaria in the western Amazon [5, 9, 11, 12].

However, it is known that mass rearing and exposure to artificial environments can influence evolutionary changes in the original population, and thereby give rise to strains that are more sensitive to stress and have higher metabolic rates or stronger biosynthetic machinery [13, 14]. Such changes in colonized populations may impact their susceptibility to infection. An evaluation of an *An. darlingi* colony from Peru showed low to moderate differentiation between field and laboratory populations after 21 generations [15], and wild and colonized *Anopheles stephensi* mosquitoes were found to be equally susceptible to *Plasmodium* infection after 66–86 generations [16].

Therefore, the aim to assess whether the mosquito colonies’ susceptibility to *P. vivax* has remained viable after 25 generations and thus whether the colonies remain good models for vector-parasite studies. To make this assessment, the susceptibility of colonized *An. darlingi* and *An. deaneorum* to *P. vivax* circulating in the Western Amazon was estimated. Susceptibility estimates were made by observing prevalence rates, infection intensity and survival of infected mosquitoes. Additionally, the correlation between gametocytaemia by microscopy and prevalence and infection intensity in these two species was assessed.

## Methods

### Blood collection from *Plasmodium vivax* patients

Study participants were recruited from patients diagnosed with vivax malaria by Giemsa-stained blood smears taken at the Centro de Pesquisa em Medicina Tropical (CEPEM), in Porto Velho, Rondonia, a malaria-endemic area in the Amazon region of Brazil.

Malaria transmission in Porto Velho shows seasonal peaks of incidence following the rainy periods (from October to April). In the last five years, Porto Velho has reported increases in malaria cases, with a mean of more than 5000 cases per year. Most cases (~ 95%) are caused by *P. vivax* [17]. From 2018 to 2020, in all periods of malaria transmission, vivax malaria patients were invited to participate in the study. Volunteers were recruited after fulfillment of the following criteria: thick blood smear positive exclusively for *P. vivax*, age between 18 and 85 years, absence of signs or symptoms of severe malaria or concomitant disease, with and without a previous history of malaria, non-pregnant and agreement to study procedures. After subjects provided informed consent, about 10 mL of blood was drawn by venipuncture and placed immediately into glass vials coated with heparin to prevent clotting. The tubes were stored in a water flask at 37 °C and transported to the Plataforma de Produção e Infecção de Vetores da Malária (PIVEM) insectary for DMFA. The decision to participate had no effect on malaria treatment and the anti-malarial chemotherapy followed the Brazilian Ministry of Health guidelines.

### *Plasmodium vivax* parasitaemia and gametocyte counts

A second thick blood smear was prepared at PIVEM, stained in Giemsa (3% stain working solution), and examined for the presence of malaria parasites under light microscopy using a 100x oil immersion lens. Counts per 200 leukocytes of the sexual (gametocyte) and asexual forms of *P. vivax* were performed and parasite density was calculated as the number of parasites/ µL by assuming a fixed leukocyte count of 8,000 leukocytes/µL [18]. Results were independently confirmed by two well-trained microscopists and inconsistencies were resolved by a senior microscopist.

### Mosquito colonies

*Anopheles darlingi* and *An. deaneorum* have been maintained in the insectary of PIVEM in Fiocruz Rondônia since 2018. The mosquitoes were reared at 26 °C ± 1 °C with a relative humidity of 70 ± 10%, and provided with 15% honey [9, 10]. Mosquitoes were fed on parasite-containing blood 3–4 days post-emergence.

### Mosquito infection experiments

Prior to DMFA, as described by Moreno et al., female mosquitoes from each colony were deprived of sucrose overnight [19]. A total of 72 *P. vivax* isolates were used for DMFA, and of these, 17 were used for paired-feeding experiments (*An. darlingi vs. An. deaneorum*), 43 were used for feeding experiments using just *An. darlingi* mosquitoes, and 12 were used for feeding experiments using just *An. deaneorum* mosquitoes. Additional table shows this in more detail in Additional file 1: Table S1.

In each DMFA, cohorts of about 40–100 mosquitoes were fed on *P. vivax* blood isolates for 30 min. Since the CEPEM clinical site is just 1 km from the PIVEM insectary, it was possible to transport the blood (maintained at 37 °C inside a water flask) and feed the mosquitoes within 5–10 min after blood collection. Two mL of heparinized blood from each volunteer were added to a 5 cm diameter, water-jacketed glass membrane feeder fitted with a slightly stretched Parafilm membrane. Blood was kept at a constant 37 °C during the mosquito feeding. Mosquitoes were allowed to feed for 30 min. After this time, the unfed and partially fed mosquitoes were removed; only fully fed mosquitoes were kept in the experimental cages for subsequent examination of sporogonic development. A cotton wool pad soaked with 15% honey solution was provided regularly and changed every other day until dissection.

The effect of infection on survival was evaluated for mosquitoes fed on infected and uninfected blood, and survival was evaluated from initial blood-feeding until sporogonic development (days 1 to 14).

### Mosquito dissections, microscopy and parasite counting

Half of the mosquitoes were dissected on day 7 post blood feeding in order to assess oocyst load in the midgut and the rest were dissected on day 14 post blood feeding in order to assess sporozoite load in the salivary glands (see Additional file 1: Tables S2–S4). Mosquitoes were anaesthetized on ice, placed in a glass beaker (50 mL) with 70% ethanol and then transferred to another glass beaker containing phosphate buffered saline (PBS 1X). The midguts were dissected in PBS 1X, stained with 0.2% commercial mercurochrome (SIGMA), placed under a glass cover, and examined for the presence of oocysts using microscopy (10 X). The salivary glands were dissected in RPMI and transferred to a tube with 15 µL of RPMI in a pool of 2 to 10 salivary glands. Subsequently, the pool was homogenized in a glass tissue grinder and then centrifuged for 30 sec. Sporozoite numbers were counted using a Neubauer chamber hematocytometer under microscope (40X).

### Statistics

Statistical analyses were performed using the GraphPad Prism v0.9.0 software. Differences between species in blood-feeding rate and prevalence were analysed using the Chi-square test. The blood-feeding rate was determined by proportion of full engorged mosquitoes after the 30 min to blood feeding and infection prevalence was determined by proportion of mosquitoes infected with oocysts at 7 dpi. Differences in median oocyst and sporozoite production (intensity of infection) were analysed using a Mann-Whitney test that included only individual mosquitoes that produced > 0 oocysts. Spearman’s r was calculated to evaluate the correlation between asexual/gametocytaemia and prevalence, and asexual/gametocytaemia and intensity of infection with a significance level of 0.05. The Kaplan-Meier survival curve was used to represent the probability of survival for mosquitoes fed on infected and uninfected blood over a 14-day follow-up.

## Results

A total of 8,427 laboratory-reared mosquitoes were processed: 2,938 mosquitoes (1,477 *An. darlingi* and 1,461 *An. deaneorum*) were used for comparative susceptibility (paired feeding) (Table 1), and 1545 mosquitoes (1273 *An. darlingi* and 272 *An. deaneorum*) were used for correlation analyses (*P. vivax* parasitaemia and infection) and survival. A total of 72 *P. vivax* infections were performed with parasitaemia varying among donors: asexual parasitaemia (180 to 27,405 asexual parasitaemia per µL, median 4200) and gametocytaemia (0 to 1320 gametocytes per µL, median 210) (Table 2). Two infections failed to produce oocysts in both species’ midgut. Data from each patient and number of mosquitoes used to each DMFA are shown in Additional file 1: Table S1.

**Table 1:** Susceptibility of *Anopheles darlingi* and *Anopheles deaneorum* to *Plasmodium vivax*

Species	Number of *P. vivax* isolates	Engorged/ Number of mosquitoes (%)	Positive for oocyst/Midgut dissected (%)	*P value* (Chi–square)	Median oocyst (Min–Max)	*P value* (Mann–Whitney test)	Number of salivary gland dissected	Median sporozoites (Min–Max)	*P value* (Mann–Whitney test)
Paired feedings									
*An. darligi*		1281/1477 (86.73)	308/445 (69.21)		7 0.5 (1–281)		411	1840 (80 − 37,800)	
vs.	17*			< 0.0001		0.0011			< 0.0001
*An. deaneorum*		862/1461 (59.00)	81/239 (33.89)		4.0 (1–224)		201	400 (80 − 13,333)	

*Two infections failed

**Table 2:** Data of the *Plasmodium vivax* isolates of the study (n = 72)

	Minimum	Maximum	Median
Age of patients (years)	18	83	37
Asexual parasites/µL	180	27,405	4200
Gametocytes/µL	0	1320	210

**Fig. 1:**
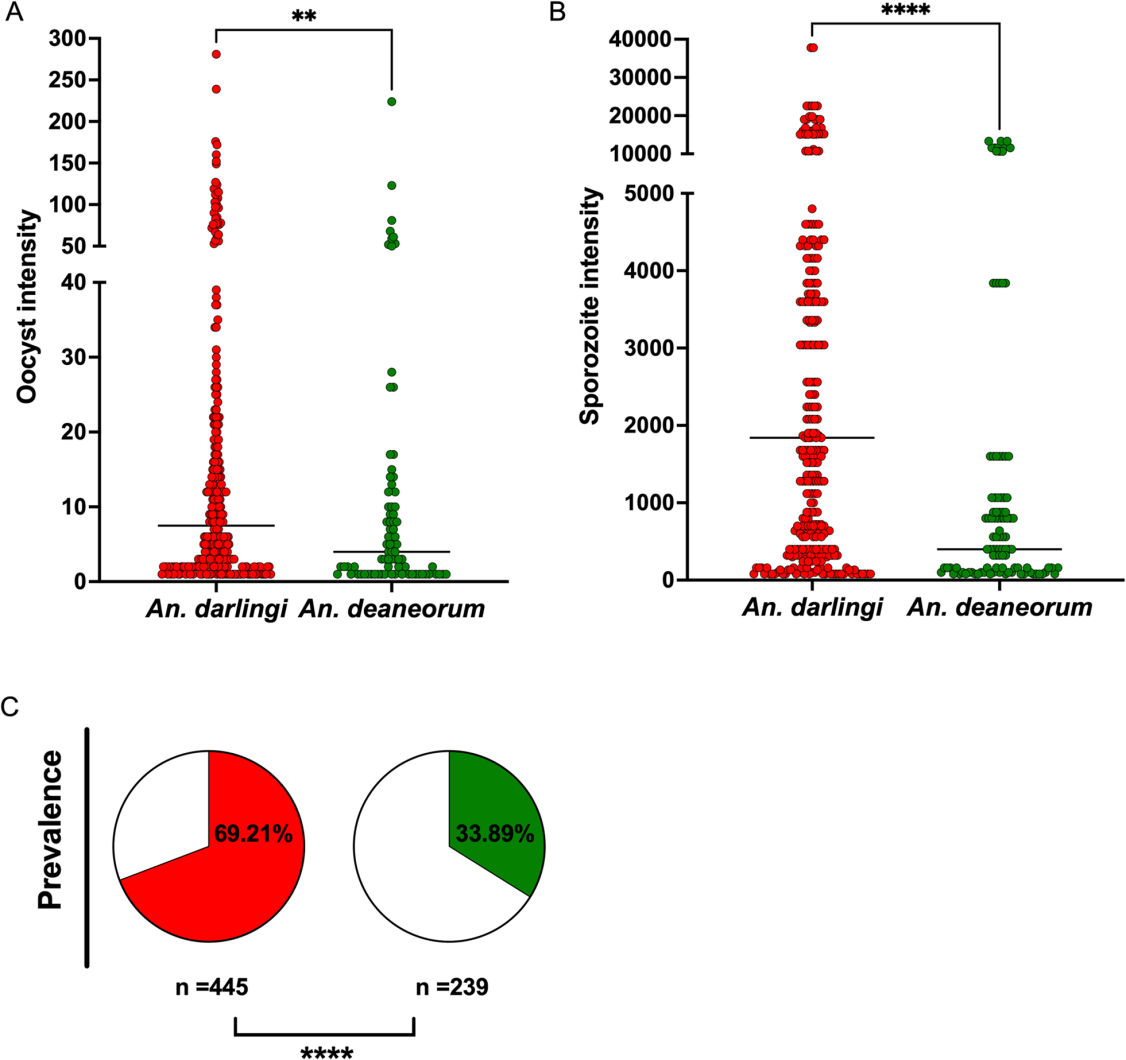
*Plasmodium vivax* infection in *Anopheles darlingi *(red colour) and *Anopheles deaneorum* (green colour). **A)** Distribution of oocyst intensity, each point represents a positive midgut. **B)** Distribution of sporozoite intensity, each point represents a positive salivary gland. Medians are indicated. Intensity defined by two-sided Mann-Whitney *U* test. **C)** Prevalence of infection is shown in the pie charts. Prevalence was defined by two-sided Chi-squared test. Asterisks indicate statistical significance. **P = 0.0011, ****P < 0.0001. The data correspond to 15 independent biological experiments

Significant differences were detected in prevalence (*p* < 0.0001), and oocyst (*p* = 0.0011) and sporozoite (*p* < 0.0001) intensity between *An. darlingi* and *An. deaneorum* (Fig. 1; Table 1). *Anopheles darlingi* showed a higher proportion of infected mosquitoes (69.21%), and higher oocyst and sporozoite intensity (median of oocysts = 7.5, range of 1–281; median of sporozoites = 1840, range of 80 − 37,720). On the other hand, *An. deaneorum* showed a mosquito infection rate of 33.89%, median oocyst production of 4.0 (range of 1–224) and median sporozoite production of 400 (range of 80–13,333) (Fig. 1; Table 1, see Additional file 1: Table S2). The *An. darlingi* susceptibility was maintained when compared to mosquitoes from the beginning of colonization (prevalence: 70 to 97%) [9], while *An. deaneorum* showed a decreased on the susceptibility (prevalence: 66 to 100%) [10].

**Fig. 2:**
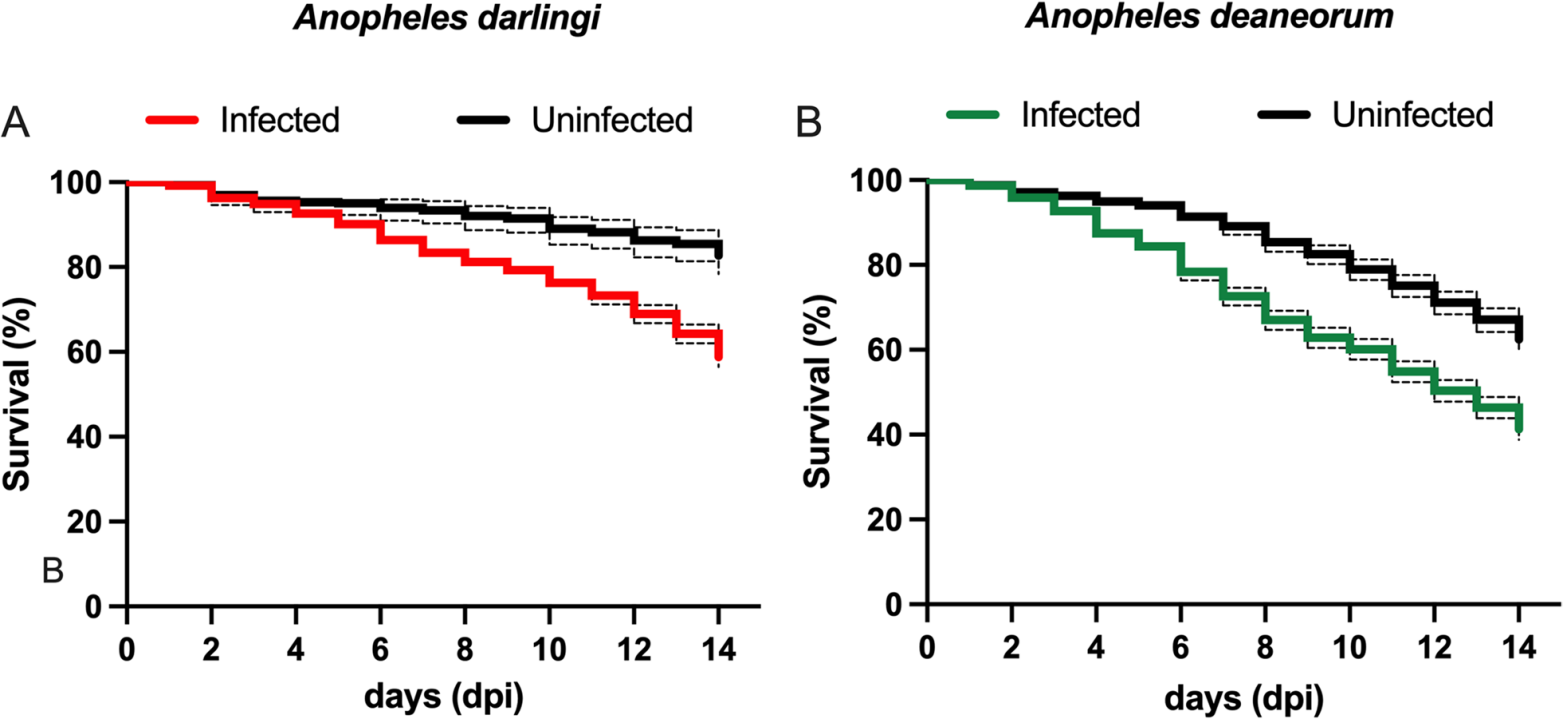
Kaplan-Meier curve of *Anopheles darlingi* (**A)** and *Anopheles deaneorum* (**B)** infected (coloured line) and uninfected (black line). Mortality was recorded in adult mosquitoes from first day to 14th day post blood feeding

The blood-feeding rate of *An. deaneorum* was less (59.00%) than that of *An. darlingi* (86.73%) (Table 1). Mosquito survival was also assessed in comparison to mosquitoes fed with healthy blood. From day 1 (blood feeding) to day 14, mortality was evaluated daily for both species in infected and uninfected groups. Overall, there was a significant difference in survival between infected and uninfected mosquitoes for both species (*p <* 0.0001). Survival rates were lower among infected mosquitoes (Fig. 2A, B).

**Fig. 3:**
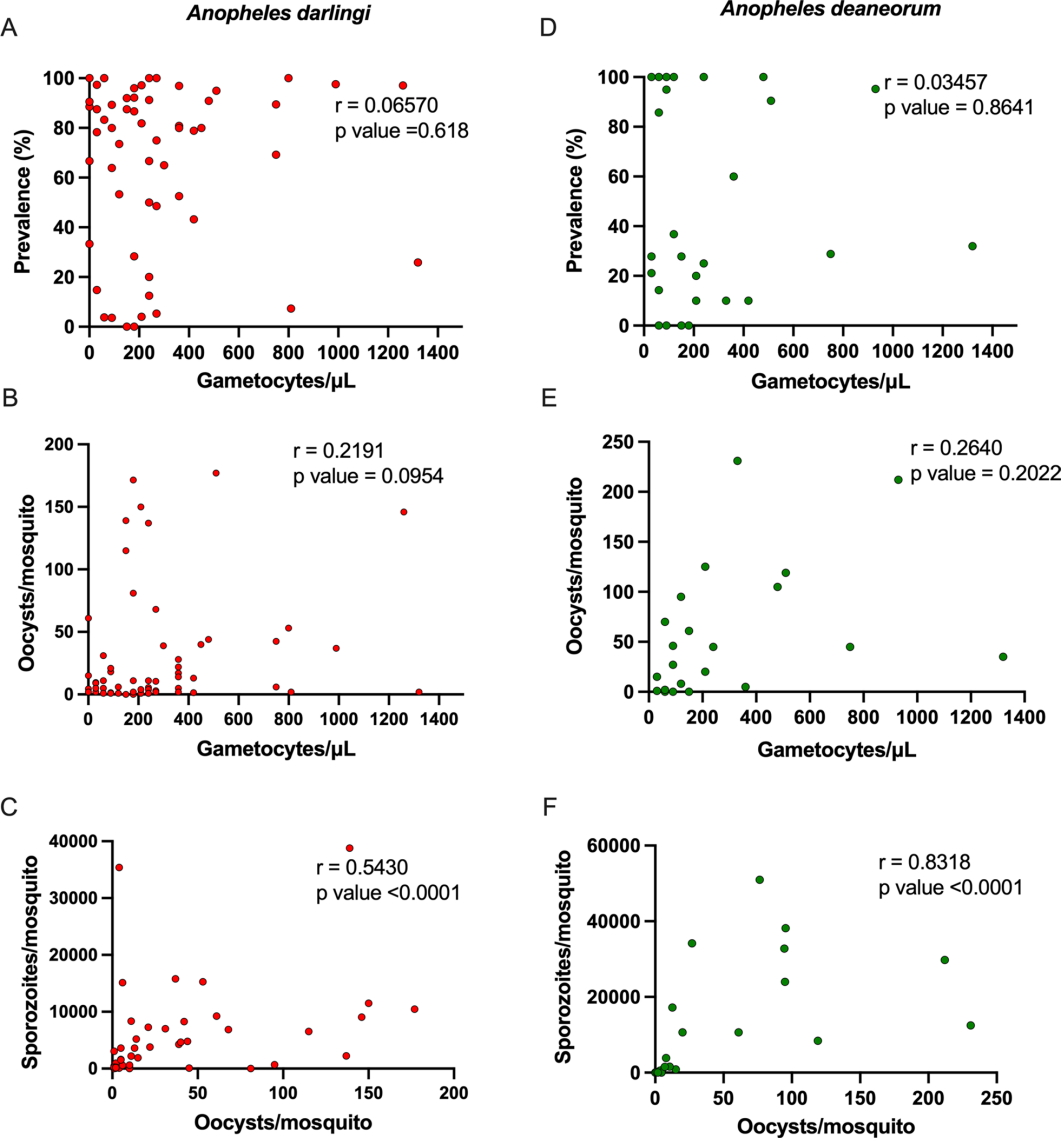
Correlation between gametocytes/µL and prevalence in *Anopheles darlingi* (**A)** and *Anopheles deaneorum* (**D**). Correlation between gametocytes/µL and oocysts/mosquito in *Anopheles darlingi* (**B)** and *Anopheles deaneorum* (**E**). Correlation between oocysts/mosquito and sporozoites/mosquito in *Anopheles darlingi* (**C)** and *Anopheles deaneorum* (**F**). Spearman’s correlation coefficient was used to evaluate the relationship between data

Microscopic examination showed that 92.9% of symptomatic patients had gametocytes circulating in the bloodstream on the day of blood collection. However, no association was found between gametocytaemia and prevalence, or gametocytaemia and infection intensity in either species (Fig. 3A, B, D, E). Blood from symptomatic patients who had no gametocytes circulating in their bloodstreams infected the mosquitoes at rates ranging from 12.5 to 100%. Furthermore, blood from symptomatic patients who had weak gametocytes (between 30 and 200 gametocytes/µL) showed mosquito infection prevalence ranging from 0.0 to 100% (Fig. 3A, D). The correlation analyses between oocysts/mosquito and sporozoites/mosquito revealed a moderate to strong correlation for both species: *An. darlingi* (r = 0.5430, p value < 0.0001) (Fig. 3C) and *An. deaneorum* (r = 0.8318, p value < 0.0001) (Fig. 3F). An additional Table shows oocyst and sporozoite numbers of each DMFA (see Additional file 1: Tables S2–S4).

## Discussion

Colonies of neotropical mosquito species are being maintained in Brazil to study the parasite-vector relationship [9, 10]. For this purpose, the *Plasmodium* susceptibility of the colonized mosquitoes needs to be maintained so that the mosquitoes can be used as models for pathogen transmission. At the beginning of colonization, the *An. darlingi* and *An. deaneorum* colonies were confirmed to be *P. vivax* susceptible [9, 10]. Furthermore, when *An. darlingi* and *An. deaneorum* susceptibility was compared in first generation mosquitoes, no difference in infection rates and infection intensity was observed [11, 20]. However, the data from generations F10 to F25 show a significant difference in prevalence and infection intensity between *An. darlingi* and *An. deaneorum*. *Plasmodium vivax* prevalence in *An. deaneorum* was less than 40%, while in *An. darlingi* was more than 60%. In mosquito *Plasmodium* susceptibility, it is important consider the interaction among the gut microbiota, the immune system and the parasite. This, because the midgut microbiota has a potential role in mosquito *Plasmodium* susceptibility [21–23]. Although *An. darlingi* and *An. deaneorum* have been colonized in the same laboratory conditions, the microbiota of each species may be different and could explain the difference of susceptibility. Studies have demonstrated that different species reared in the same insectary may be host of a different midgut microbiota composition [24, 25]. Investigation to understand the difference in parasite-vector interaction of these two species is necessary.

Another possible explanation for the decreased susceptibility of *An. deaneorum* is the low blood-feeding rate and the high mortality registered in these *An. deaneorum* generations. The blood-feeding rate of the *An. deaneorum* mosquitoes was also less than 60% and their survival drastically decreased after 7 dpi. Although a survival decrease was also observed in the *An. darlingi* mosquitoes, more than 50% of the infected *An. deaneorum* mosquitoes were dead at 14 dpi. For efficient parasite transmission, mosquitoes need to survive long enough to allow *Plasmodium* oocyst development and subsequent salivary gland invasion by sporozoites. The cost of *Plasmodium* infection to mosquito survival is still a subject of intense debate [26, 27]. The data show a significant difference in survival between infected and uninfected mosquitoes of both species: survival rates were higher among the uninfected. However, this effect could be a relationship with the act of *Plasmodium* or even in part associated with the act of blood feeding itself. In future, it will be of interest to add unfed mosquitoes to compare with infected and uninfected feeding mosquitoes.

The impact of *P. vivax* on insect vector survival has been little investigated. Gamage-Mendis et al. showed that *P. vivax* parasitism did not appear to affect *Anopheles tessellatus* survival [28], and Andolina et al. found no correlation between *Anopheles cracens* survival and sporozoite load [29]. However, mosquito survival may be reduced by tissue damage caused during *Plasmodium* development and migration from the midgut to the salivary glands, and by the activation of a costly immune response [26, 30]. Previous studies found that elevated gametocytaemia (or parasitaemia) may also increase vector mortality [26, 31].

Mosquitoes are infected when they ingest mature gametocytes circulating in the peripheral blood [4]. Therefore, in theory, the density of *Plasmodium* gametocytes could be used to predict the infection level of *Anopheles* infected via membrane feeding assay; however, the correlation between *P. vivax* gametocytaemia and mosquito infections has proven to be variable [32–37]. In both *An. darlingi* and *An. deaneorum*, no correlation was found between blood gametocyte density and prevalence, or blood gametocyte density and midgut parasite infection load. The data showed that, even with high gametocyte density, the proportion of infected mosquitoes was low in the DMFAs, as well as low gametocyte density showed high infection rate. The data suggest that gametocyte density could not be a good predictor of mosquito infection.

Gametocytes are sometimes undetectable by microscopy [38]. Using real-time reverse transcriptase PCR gametocytaemia, Bharti et al. found a weak correlation between gametocyte density and percentage of mosquitoes infected [34]. There was also a correlation between smear and real-time reverse transcriptase PCR gametocytaemia. Regardless, there is a consensus that gametocyte density is not the only factor affecting the proportion of infected mosquitoes. Parasite genetic variation, human immune response, the maturation and the sex ratio of gametocytes and midgut microbiota composition of vectors, may also be involved, and should be considered [4, 6, 21, 39–41]. Future studies are required to better understand *P. vivax*-mosquito interactions, especially studies that identify what parameters are necessary to improve DMFA. Criteria that predict mosquito infection rates are important because they can help to optimize the mosquito screening process in accord with the experimental specifications used by infection platform.

Positive correlation between the median number of oocysts and sporozoite loads are a criterion that could be used for screening mosquito batches before sporozoite challenge studies. The positive correlation between oocyst level and sporozoite loads has been identified previously in *An. tessellatus* [28] and *Anopheles albimanus* [36] infected with *P. vivax*. Thus, improved screening will allow increase in oocyst production, and consequently, produce more sporozoites.

Currently, there is interest in a whole-parasite vaccine strategy, which requires sporozoite production in live mosquitoes [42]. Vaccine development for vivax malaria is underway and a model to assess vaccine efficacy is urgently needed [36]. One of the aims of PIVEM is to develop a model for assessing potential vaccine candidates. Thus, optimal conditions for mosquito probing and infection are an important requirement for studies that model *P. vivax*- *Anopheles* interaction.

## Conclusions

This study confirms that *An. darlingi* colony from the Brazilian Amazon remains highly susceptible to *P. vivax* infection, and thereby demonstrates that *An. darlingi* is an excellent species for modelling pathogen transmission. On the other hand, *An. deaneorum* could serve as a model for immunity studies due the low susceptibility under current colonized conditions. In DMFA, gametocytaemia determined by light microscopy is not a good criterion for predicting mosquito infection by *P. vivax*, and other predictive factors should be investigated. Finally, the data show that oocyst intensity should be used for scheduling sporozoite experiments.

## Supplementary Information

**Additional file 1: Table S1.** Data from each patient and mosquitoes number used to DMFA. **Table S2.** Oocyst and sporozoites number of each DMFA from paired feeding assay. **Table S3.** Oocyst and sporozoites number of each DMFA using only *Anopheles darlingi. ***Table S4.**Oocyst and sporozoites number of each DMFA using only *Anopheles deaneorum*.

## Data Availability

All relevant data are within the manuscript and its supporting information files.

## Abbreviations

DMFA: Direct Membrane-feeding Assay
CEPEM: Centro de Pesquisa em Medicina Tropical
PIVEM: Plataforma de Produção e Infecção de Vetores da Malária
TBV: Transmission blocking vaccine
